# Structures of New Phenolics Isolated from Licorice, and the Effectiveness of Licorice Phenolics on Vancomycin-Resistant *Enterococci*

**DOI:** 10.3390/molecules190913027

**Published:** 2014-08-25

**Authors:** Eerdunbayaer, Mohamed A. A. Orabi, Hiroe Aoyama, Teruo Kuroda, Tsutomu Hatano

**Affiliations:** 1 Department of Natural Product Chemistry, Okayama University Graduate School of Medicine, Dentistry and Pharmaceutical Sciences, Tsushima-naka, Kita-ku, Okayama 700-8530, Japan; E-Mails: eerdun3@163.com (E.); m_a_orabi@yahoo.com (M.A.A.O.); ph20101@s.okayama-u.ac.jp (H.A.); 2 Faculty of Pharmacy, Al-Azhar University, Assiut 71524, Egypt; 3 Drug Discovery and Technology Center, Okayama University Graduate School of Medicine, Dentistry and Pharmaceutical Sciences, Tsushima-naka, Kita-ku, Okayama 700-8530, Japan; E-Mail: tkuroda@cc.okayama-u.ac.jp

**Keywords:** licorice, *Glycyrrhiza uralensis*, flavonoid, 2-aryl-3-methylbenzofuran, VRE, antibacterial effect, HPLC

## Abstract

Licorice, which is the underground part of *Glycyrrhiza* species, has been used widely in Asian and Western countries as a traditional medicine and as a food additive. Our continuous investigation on the constituents of roots and stolons of *Glycyrrhiza uralensis* led to the isolation of two new phenolics, in addition to 14 known compounds. Structural studies including spectroscopic and simple chemical derivatizations revealed that both of the new compounds had 2-aryl-3-methylbenzofuran structures. An examination of the effectiveness of licorice phenolics obtained in this study on vancomycin-resistant strains *Enterococcus faecium* FN-1 and *Enterococcus faecalis* NCTC12201 revealed that licoricidin showed the most potent antibacterial effects against both of *E. faecalis* and *E. faecium* with a minimum inhibitory concentration (MIC) of 1.9 × 10^−5^ M. 8-(γ,γ-Dimethylallyl)-wighteone, isoangustone A, 3'-(γ,γ-dimethylallyl)-kievitone, glyasperin C, and one of the new 3-methyl-2-phenylbenzofuran named neoglycybenzofuran also showed potent anti-vancomycin-resistant *Enterococci* effects (MIC 1.9 × 10^−5^–4.5 × 10^−5^ M for *E. faecium* and *E. faecalis*). The HPLC condition for simultaneous detection of the phenolics in the extract was investigated to assess the quality control of the natural antibacterial resource, and quantitative estimation of several major phenolics in the extract with the established HPLC condition was also performed. The results showed individual contents of 0.08%–0.57% w/w of EtOAc extract for the major phenolics in the materials examined.

## 1. Introduction

Infectious diseases caused by multidrug-resistant bacteria, including methicillin-resistant *Staphylococcus aureus* (MRSA) and vancomycin-resistant *Enterococci* (VRE) are serious problems worldwide [1]. Although *Enterococcus* bacteria are considered ordinary components in the healthy human intestinal flora, they are responsible for complicated urinary tract infections and serious endocarditis [2]. *Enterococcus faecium* and *Enterococcus faecalis* account for >95% of *Enterococcus* isolates from clinical cultures [3], and only a few drugs such as linezolid and a combination of quinupristin and dalfopristin are used clinically for VRE [4]. Since the adverse effects of these drugs have been revealed and drug resistance to them may appear soon, the development of a new group of low toxicity antibacterial agents is needed. Licorice has been used as a food sweetener and is one of the oldest and most frequently used crude drugs in traditional medicine, particularly in Asian countries. A variety of pharmaceutical functions, such as antiulcer, anti-inflammatory, antiviral, and anticarcinogenic activities have been reported for licorice constituents [5,6,7,8], and the antibacterial effects of licorice phenolics have been demosntrated for various bacterial species [9,10,11,12,13,14]. The effect of a compound isolated from licorice, gancaonin I (**1**), on VRE was also demonstrated in a previous study [15].

Our continuous studies have revealed the antibacterial effects of several licorice phenolics on MRSA, particularly those with both γ,γ-dimethylallyl (prenyl) and hydroxyl groups [16]. Licoricidin (**2**) has the same structural features and displays a suppressive effect on oxacillin resistance shown by MRSA [16]. We also reported the anti-VRE effects of several licorice phenolics in a previous study [17]. Our further investigations have led to the isolation of 16 phenolic compounds including two new compounds with rarely occurring 2-aryl-3-methylbenzofuran structures. This paper explains the structural determination of the new compounds and the effects of those phenolics on two VRE stains. In addition, an analytical condition for high-performance liquid chromatography (HPLC) to simultaneously analyze polyphenolic constituents in the EtOAc extract was established for quality control of the antibacterial resource, and several major phenolics in the extract were quantitated using the established HPLC condition.

## 2. Results and Discussion

**Figure 1:**
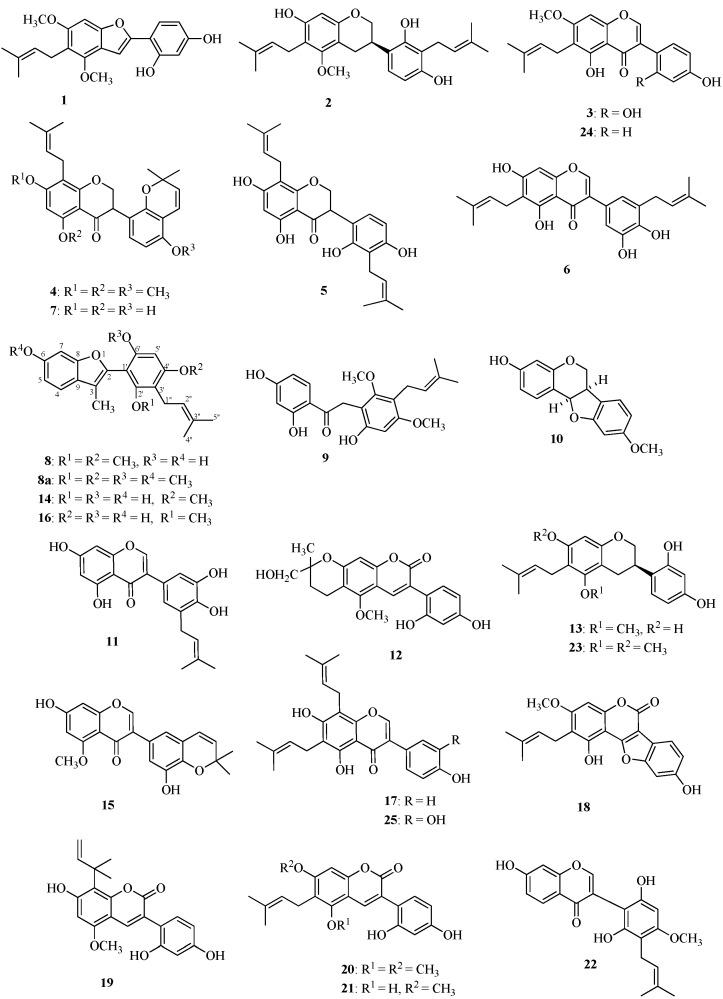
Structures of the compounds **1**–**25** found in the EtOAc extract of *Glycyrrhiza* *uralensis* roots and stolons (Tohoku licorice). Structures **2**–**17** are the compounds isolated in this study ^a^.

^a^ The configuration at C-3 in each of compounds **4**, **5**, and **7** was not determined in the previous papers [20,21].

A part of the EtOAc extract obtained from powdered licorice was subjected to column chromatography on ODS-gel and eluted with increasing concentrations of MeOH in H_2_O and then with increasing concentrations of CHCl_3_ in MeOH. The eluate with 50% CHCl_3_ in MeOH was subjected to column chromatography on MCI-gel CHP-20P with increasing concentrations of MeOH in H_2_O. Fractions from the column were purified by preparative HPLC, to give 16 licorice phenolics, including licoricidin (**2**) [18], 7-*O*-methylluteone (**3**) [19], glyasperin J trimethyl ether (**4**) [20], 3'-(γ,γ-dimethylallyl)-kievitone (**5**) [21], isoangustone A (**6**) [22], glyasperin J (**7**) [20], compound A (**8**), licoriphenone (**9**) [23], demethylhomopterocarpan (**10**) [24], glycyrrhisofavone (**11**) [25], licopyranocoumarin (**12**) [26], glyasperin C (**13**) [27], compound B (**14**), glycyrrhiza-isofavone B (**15**) [28], glycybenzofuran (**16**) [29], and 8-(γ,γ-dimethylallyl)-wighteone (**17**) [30] from the respective fractions. The structures of **2**–**7**, **9**–**13**, and **15**–**17** (Figure 1) were identified by comparisons of spectral data with values reported in the literature [18,19,20,21,22,23,24,25,26,27,28,29,30], whereas the two arylbenzofurans, temporarily named compounds A (**8**) and B (**14**) (Figure 1), are new, and their structure elucidations are described below.

### 2.1. Structures of the New Compounds

Compound A (**8**): This compound was obtained as a light brown powder. Its molecular formula was C_22_H_24_O_5_, based on the [M + H]^+^ ion peak in the high-resolution fast-atom bombardment mass spectrometry (HR-FAB-MS). The ultraviolet (UV) spectrum of **8** showed absorption maxima at 214 (log ε 4.11), 238 (4.01), and 305 nm (4.52), indicating structural similarity to those of the known compounds glycybenzofuran (**16**) and licocoumarone (**18**) with 2-arylbenzofuran skeletons. The ^1^H nuclear magnetic resonance (NMR) spectrum of **8** (in acetone-*d*_6_) showed resonances of three aromatic protons at δ_H_ 7.30 (d, *J* = 8.4 Hz, H-4), 6.87 (d, *J* = 2.4 Hz, H-7), and 6.77 (dd, *J* = 2.4, 8.4 Hz, H-5), forming an ABX spin system, and a one-proton singlet at δ_H_ 6.41 (H-5'). The spectrum also showed four sets of proton resonances at δ_H_ 5.16 (1H, t, *J* = 6.6 H_Z_, H-2"), 3.32 (2H, d, *J* = 6.6 Hz, H-1"), 1.60 (3H, s), and 1.70 (3H, s) (2 × CH_3_ at C-3"), which are assignable to those of a γ,γ-dimethylallyl (prenyl) group. In addition, proton resonances characteristic of two methoxyl groups at δ_H_ 3.82 and 3.35 (3H each, s) and one methyl group at δ_H_ 1.89 (3H, s) were seen in the aliphatic region of the spectrum.

The ^13^C-NMR spectrum showed six carbon resonances due to oxygenated *sp*^2^ carbons (δ_C_ 160.0, 158.9, 156.2, 155.8, 153.1, and 145.7) and eight carbon resonances attributable to non-oxygenated *sp*^2^ carbons [δ_C_ 123.0, 119.6, 114.2 (2C) 111.5, 102.1 97.9, and 96.6]. The spectrum also showed five carbon resonances due to the prenyl group (δ_C_ 17.2, 22.5, 25.3, 124.3, and 129.9) and two methoxyl groups (δ_C_ 60.7 and 55.0). In addition to these resonances, the spectrum showed a methyl carbon resonance (δ_C_ 7.9), ascribable to the methyl group at C-3 of the 2-arylbenzofuran structure.

**Figure 2:**
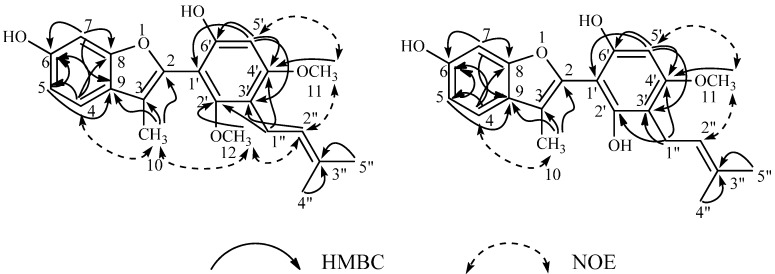
HMBC and NOESY correlations observed for compounds **8** and **14**.

The assignments of these proton and carbon resonances were substantiated by the heteronuclear single quantum correlation (HSQC) and heteronuclear multiple-bond correlation (HMBC) spectral data as summarized in Table 1. Key HMBC correlations among them and the nuclear Overhauser effect spectroscopy (NOESY) correlations indicating the locations of the respective substituents on the 2-arylbenzofuran skeleton are shown in Figure 2.

**Table 1:** ^1^H- and ^13^C-NMR assignments, HSQC and HMBC correlations for compounds (**8**) and (**14**) (600 MHz for ^1^H and 151 MHz for ^13^C, acetone-*d*_6_, 27 °C) ^a,b^.

Position	4'-*O*-Methylglycybenzofuran (8)				Neoglycybenzofuran (14)			
	δ_C_	HSQC ^a^	δ_H_ ( *J* in Hz)	HMBC ^b^	δ_C_	HSQC ^a^	δ_H_ ( *J* in Hz)	HMBC ^b^
C-2	145.7	C		H-10	145.5	C		H-10
C-3	114.2	C		H-10	114.4	C		H-10
C-4	119.6	CH	7.30, d (8.4)		119.3	CH	7.23, d (9.0)	
C-5	111.5	CH	6.77, dd (2.4, 8.4)	H-4	111.4	CH	6.71, dd (2.4, 9.0)	
C-6	155.8	C		H-5, 7	155.7	C		H-4, 5, 7
C-7	97.9	CH	6.87, d (2.4)		97.8	CH	6.82, d (2.4)	
C-8	156.2	C		H-4, 7	156.2	CH		H-4, 7
C-9	123.0	C		H-5, 7, 10	123.8	CH		H-5, 7, 10
C-10	7.9	CH_3_	1.89 s		8.7	CH_3_	1.97 s	
C-1'	102.1	C		H-5'	103.5	C		H-5'
C-2'	158.9	C		H-1"	159.1	C		H-1"
C-3'	114.2	C		H-5', H-2"	112.9	C		H-5', 1", 2"
C-4'	160.0	C		H-5', H-1"	160.0	C		H-5', 1"
C-5'	96.6	CH	6.41 s		98.7	CH	6.28 s	
C-6'	153.1	C		H-5'	155.7	C		H-5'
C-1"	22.5	CH_2_	3.32, d (6.6)		22.9	CH_2_	3.13, d (6.6)	
C-2"	124.3	CH	5.16, t (6.6)		124.6	CH	5.12, t (6.6)	
C-3"	129.9	C		H-4", H-5"	129.7	C		H-4", 5"
C-4"	17.2	CH_3_	1.60 s		17.8	CH_3_	1.69 s	
C-5"	25.3	CH_3_	1.70 s		25.5	CH_3_	1.63 s	
-OCH_3_	60.7	CH_3_	3.82 s	H-11	60.6	CH_3_	3.28 s	H-11
-OCH_3_	55.0	CH_3_	3.35 s	H-12				

^a^ HSQC, shows the relationship between a proton directly connected to a carbon. ^b^ HMBC correlations, optimized for 5 Hz, are from proton(s) stated to the optimized carbons.

The locations of the hydroxyl and methoxyl groups, and the prenyl group on the B-ring of this compound were shown by the following HMBC correlations. Correlation of the methylene proton resonance at δ_H_ 3.32 (H-1") of the prenyl moiety and an -OCH_3_ resonance (δ_H_ 3.82) with a common oxygenated aromatic carbon resonance at δ_C_ 160.0 (C-4') and also the correlations of the same methylene proton resonance (H-1") and a methoxyl proton resonance (δ_H_ 3.35) with a common oxygenated carbon resonance at δ_C_ 158.9 (C-2'). The carbon resonance at δ_C_ 160.0 (C-4') was also correlated with the singlet proton resonance at δ_H_ 6.41 (H-5', directly correlated with the C-5 carbon resonance at δ_C_ 96.6 in the HSQC spectrum), and this proton resonance was also correlated with an oxygenated aromatic carbon at δ_C_ 153.1 (C-6'), which was not correlated with any methoxyl proton resonance. The H-5' resonance was also correlated with the carbon resonances at δ_C_ 114.2 (C-3') and δ_C_ 102.1 (C-1') of the same aromatic ring. The last two carbon resonances were discriminated by a correlation of the methylene proton resonance (H-1") to the carbon resonance at δ_C_ 114.2 (C-3'). The sequence C-1' (δ_C_ 102.1)–C-2' (δ_C_ 158.9, with a methoxyl group)–C-3' (δ_C_ 114.2, with the prenyl group)–C-4' (δ_C_ 160.0, with a methoxyl group)–C-5' (δ_C_ 96.6)–C-6' (δ_C_ 153.1) was thus assigned for the B-ring. In addition, the NOESY spectrum of this compound showed correlations of the methine proton resonance at δ_H_ 5.16 (H-2" of the prenyl moiety) with the two methoxyl resonances at δ_H_ 3.82 (-OCH_3_ at C-4') and δ_H_ 3.35 (-OCH_3_ at C-2'), and the former methoxyl resonance also showed a correlation with the proton resonance at δ_H_ 6.41 (C-5'), in agreement with the sequence described above.

The presence of a methyl group at C-3 was clearly indicated by the HMBC correlations of the methyl proton resonance at δ_H_ 1.89 (H-10) with the carbon resonance at δ_C_ 145.7 (C-2), 114.2 (C-3), and 123.0 (C-9). The NOESY correlations of this proton resonance with the methoxyl proton resonance at δ_H_ 3.35 (-OCH_3_ at C-2'), and with the aromatic proton resonance at δ_H_ 7.30 (H-4), are also consistent with the location C-3 of the methyl group. The coupling patterns of H-4 [δ_H_ 7.30 (d, *J* = 8.4 Hz)], H-5 [δ_H_ 6.77 (dd, *J* = 2.4, 8.4 Hz)], and H-7 [δ_H_ 6.87 (d, *J* = 2.4 Hz)] resonances, forming an ABX system, indicated the location C-6 for the hydroxyl group. These data, and also the remaining HMBC correlations, satisfied the 2-aryl-3-methyl-6-hydroxybenzofuran structure.

Because structure **8** assigned to compound A was an analog of a compound reported previously, glycybenzofuran (**16**), the corresponding methylated products of compounds **8** and **16** were compared. As a result, product **8a** from **8** was the same as that obtained by methylation of **16**, as expected. The structure of compound A was thus substantiated to be 4'-*O*-methylglycybenzofuran (**8**).

Compound B (**14**): Compound B was obtained as a light brown powder. The molecular formula C_21_H_22_O_5_, which was the same as that of glycybenzofuran (**16**), was detected by HR-FAB-MS. The UV spectrum of **14** (in MeOH) showed absorption maxima at 210 (log ε 4.09), 238 (4.21), and 300 nm (4.30), where the spectral feature characteristic of the 2-arylbenzofuran skeleton was seen in the spectra of compounds **8** and **16**. The aromatic region of the ^1^H-NMR spectrum of **14** (in acetone-*d*_6_) showed a one-proton singlet at δ_H_ 6.28 and the three proton resonances forming an ABX spin system at δ_H_ 7.23 (d, *J* = 8.4 Hz, H-4), 6.82 (d, *J* = 2.4 Hz, H-7), and 6.71 (dd, *J* = 2.4, 8.4 Hz, H-5). The spectrum also exhibited characteristic resonances of a prenyl moiety at δ_H_ 3.13 (2H, d, *J* = 6.6 Hz, H-1"), 5.12 (1H, t, *J* = 6.6 Hz, H-2"), 1.69 (3H, s), and 1.63 (3H, s) (*gem*-dimethyl at C-3"). In addition, the spectrum exhibited a methyl proton resonance at δ_H_ 1.97 (3H, s) and a methoxyl resonance at δ_H_ 3.28 (3H, s). These data indicate structural similarity of **14** to that of **8** except for the number of the methoxyl resonances. That is, **14** had a structure isomeric to **16**, concerning the placement of the methoxyl group.

The ^13^C-NMR spectrum of **14** showed resonances of 14 *sp*^2^ carbons attributable to the 2-arylbenzofuran skeleton composed of six oxygenated carbons [δ_C_ 160.0, 159.1, 156.2, 155.7 (2C), and 145.5] and eight non-oxygenated carbons (δ_C_ 123.8, 119.3, 114.4, 112.9, 111.4, 103.5, 98.7, and 97.8). The spectrum also showed a methyl carbon resonance at δ_C_ 8.7, a methoxyl carbon resonance at δ_C_ 60.6, and five carbon resonances due to a prenyl unit (δ_C_ 17.8, 22.9, 25.5, 124.6, and 129.7).

The HMBC spectrum (Table 1 and Figure 2) showed correlations δ_C_ 159.1 (C-2')–δ_H_ 3.13 (H-1" of prenyl at C-3')–δ_C_ 160.0 (C-4')–δ_H_ 3.28 (OCH_3_ at C-4'), and also the correlations δ_C_ 160.0 (C-4')–δ_H_ 6.28 (H-5')–δ_C_ 155.7 (C-6'), and δ_H_ 6.28 (H-5')–δ_C_ 103.5 (C-1'). Furthermore, the NOESY spectrum showed correlations δ_H_ 5.12 (H-2" of prenyl at C-3')–δ_H_ 3.28 (-OCH_3_ at C-4')–δ_H_ 6.28 (H-5') (Figure 2). These correlations clearly indicate the sequence C-1'–C-6' (with -OH)–C-5'–C-4' (with -OCH_3_)–C-3' (with prenyl)–C-2' (with -OH) of the B-ring structure.

The presence of the methyl group at C-3 was indicated by the HMBC correlations from the methyl proton resonance (H-10) at δ_H_ 1.97 with C-2 (δ_C_ 145.5), C-3 (δ_C_ 114.4), and C-9 (δ_C_ 123.8) and the NOESY correlations between the methyl resonance at δ_H_ 1.97 and H-4 at δ_H_ 7.23. The resonances of H-4, H-5, and H-7, forming an ABX system as shown by the ^1^H-^1^H COSY spectrum, indicated the location of a hydroxyl group at C-6, and the HMBC correlations (Figure 2) concerning these aromatic proton resonances also satisfied the location C-6 of the hydroxyl group. Based on these findings, structure **14**, which was isomeric to **16**, was assigned to compound B which accordingly was named neoglycybenzofuran. Methylation of **14** afforded **8a** and thus substantiated the structure **14** for neoglycybenzofuran.

### 2.2. Antibacterial Effects of Licorice Phenolics on VRE

The antibacterial effects of the licorice phenolics on the two species of VRE, *E. faecium* FN-1 and *E. faecalis* NCTC 12201, were estimated using the liquid dilution method as described previously [17]. The results summarized in Table 2 reveal that almost all of the licorice phenolics examined showed antibacterial effects [minimum inhibitory concentration (MIC), 1.9 × 10^−5^–3.5 × 10^−4^ M] on the VRE strains, and several ones among them showed noticeable anti-VRE effects (Table 2).

**Table 2:** Antibacterial effects of licorice phenolics on *Enterococci* (estimated minimum inhibitory concentrations, MIC) ^a^.

Compounds	Number of -OH Groups	Number of Prenyl Groups	MIC (10^−5^ M)		
			*Enterococcus faecium* FN-1		*Enterococcus faecalis* NCTC12201
**Isoflavones**					
7-*O*-Methylluteone (**3**)	3	1	8.7		8.7
Isoangustone A (**6**)	4	2	3.8		3.8
Glycyrrhisofavone (**11**)	4	1	9.0		9.0
Glycyrrhiza-isoflavone B (**15**)	2	0	35		35
8-(γ,γ-Dimethylallyl)-wighteone (**17**)	3	2	1.9		3.8
Glicoricone (**22**) ^a^	3	1	>35		>35
6,8-Diprenylorobol (**25**) ^a^	4	2	30		30
**Isoflavans**					
Licoricidin (**2**)	3	2	1.9		1.9
Glyasperin C (**13**)	3	1	4.5		4.5
**Isoflavanones**					
Glyasperin J trimethyl ether (**4**)	0	1	14		14
3'-(γ,γ-Dimethylallyl)-kievitone (**5**)	4	2	3.8		3.8
Glyasperin J (**7**)	3	1	7.5		7.5
**3-Arylcoumarins**					
Licopyranocoumarin (**12**)	2	0	>33		33
Glycyrin (**20**) ^a^	2	1	4.2		8.4
Glycycoumarin (**21**) ^a^	3	1	4.3		4.3
**Coumestans**					
Glycyrol (**18**) ^a^	2	1	35		>35
**Pterocarpans**					
Demethylhomopterocarpan (**10**)	1	0	12		12
**2-Aryl-3-methylbenzofurans**					
Gancaonin I (**1**) ^a^	2	1		4.5	4.5
4'-*O*-Methylglycybenfuran (**8**)	2	1		8.7	8.7
Noeglycybenzofuran (**14**)	3	1		4.5	4.5
Glycybenzofuran (**16**)	3	1		18	18
**Benzylphenylketones**					
Licoriphenone (**9**)	3	1		>34	34
**Standard antibacterial agents**					
Vancomycin ^a^				>6.9	>6.9
Linezolid ^a^				0.74	0.74
**EtOAc extract from Tohoku licorice**				16 µg/mL	32 µg/mL

^a^ Data taken from [17].

Among these compounds, licoricidin (**2**) (isoflavan) showed the most potent effects against both *E. faecalis* and *E. faecium* (MIC, 1.9 × 10^−5^ M). 8-(γ,γ-Dimethylallyl)-wighteone (**17**) (isoflavone), isoangustone A (**6**) (isoflavone), 3'-(γ,γ-dimethylallyl)-kievitone (**5**) (isoflavanone), glyasperin C (**13**) (isoflavan), and neoglycybenzofuran (**14**) (new, 2-aryl-3-methylbenzofuran) also showed anti-VRE effects with MICs of 1.9 × 10^−5^–4.5 × 10^−5^ M, respectively. All of these compounds have three or more phenolic hydroxyl groups and at least one prenyl group. In contrast, several other compounds such as licoriphenone (**9**) showed relatively weaker effects, and they had analogous structural features. Further experiments are required to clarify the structural factors responsible to the antibacterial effects.

The antibacterial effects of the EtOAc extract were comparable to those of potent anti-VRE constituents. Potential synergy and/or additive effects between the purified phenolics remain to be determined.

### 2.3. HPLC Analyses of Anti-VRE Phenolics for the Evaluation of EtOAc Extract from G. uralensis as a Source of Antibacterial Agent

Because of the important uses of licorice in traditional medicine, qualitative and quantitative analyses of licorice constituents and licorice products have been reported [31,32,33,34,35,36]. Remarkable anti-VRE effects of several licorice phenolics shown in our current and previous studies [17] suggested requirements of the identification and quantitation of those constituents. We therefore developed an HPLC-UV method for the simultaneous detection of major isolated phenolic constituents in EtOAc extract from *G. uralensis*, in order to evaluate the quality of the extract as a source of antibacterial agent.

**Figure 3:**
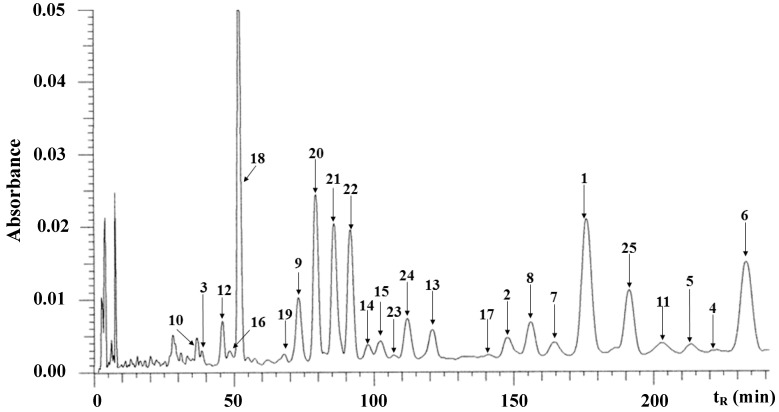
HPLC-UV chromatogram of *G. uralensis* (Tohoku licorice) EtOAc extract at 280 nm ^a−c^.

^a^ Column, YMC-Pack Pro C18 (6.0 mm i.d. × 150); mobile phase, H_2_O/MeCN/MeCOOH (55:40:5, v/v/v); flow rate, 1.0 mL/min; oven temperature, 40 °C; detector, Hitachi L2455. ^b^ The numbers represent the isolated compounds, as displayed in Figure 1. ^c^ The configuration of each of the optically active constituents was not reflected in the present analytical conditions.

The HPLC-UV profile of the EtOAc extract from Tohoku licorice used in the present study under the established condition is shown in Figure 3. Each constituent in the HPLC profile was identified by comparisons of its retention time, UV and MS spectra (data not shown) with those of the isolated one. The elution order of the identified constituents was as follows: demethylhomopterocarpan (**10**, *t*_R_ 38.6 min), 7-*O*-methylluteone (**3**, *t*_R_ 41.2 min), licopyranocoumarin (**12**, *t*_R_ 46.0 min), glycybenzofuran (**16**, *t*_R_ 46.6 min), glycyrol (**18**, *t*_R_ 52.9 min), licoarylcoumarin (**19**, *t*_R_ 68.0 min), licoriphenone (**9**, *t*_R_ 73.2 min), glycyrin (**20**, *t*_R_ 79.4 min), glycycoumarin (**21**, *t*_R_ 85.9 min), glicoricone (**22**, *t*_R_ 91.8 min), neoglycybenzofuran (**14**, *t*_R_ 98.1 min), glycyrrhiza-isofavone B (**15**, *t*_R_ 102.4 min), glyasperin D (**23**, *t*_R_ 107.4 min), gancaonin G (**24**, *t*_R_ 112.1 min), glyasperin C (**13**, *t*_R_ 121.0 min), 8-(γ,γ-dimethylallyl)-wighteone (**17**, *t*_R_ 141.1 min), licoricidin (**2**, *t*_R_ 147.9 min), 4'-*O*-methylglycybenzofuran (**8**, *t*_R_ 156.1 min), glyasperin J (**7**, *t*_R_ 164.5 min), gancaonin I (**1**, *t*_R_ 176.1 min), 6,8-diprenylorobol (**25**, *t*_R_ 191.3 min), glycyrrhisofavone (**11**, *t*_R_ 202.9 min), 3'-(γ,γ-dimethylallyl)-kievitone (**5**, *t*_R_ 213.5 min), glyasperin J trimethyl ether (**4**, *t*_R_ 222.8 min), and isoangustone A (**6**, *t*_R_ 233.2 min).

Quantitative analysis of several compounds was performed under the same HPLC condition, and the amounts of the major phenolic constituents are shown in Table 3. Among these major phenolics, gancaonin I (**1**) and isoangustone A (**6**) showed potent anti-VRE effects.

**Table 3:** Contents of major licorice phenolics in *G. uralensis* (Tohoku licorice) EtOAc extract.

Compound	Content (% w/w) ^a^
Glycyrol (**18**)	0.54 ± 0.036
Gancaonin I (**1**)	0.49 ± 0.025
Isoangustone A (**6**)	0.34 ± 0.031
Glycyrin (**20**)	0.26 ± 0.015
Glycycoumarin (**21**)	0.24 ± 0.010
Glicoricone (**22**)	0.18 ± 0.023
6,8-Diprenylorobol (**25**)	0.094 ± 0.013
Licoriphenone (**9**)	0.082 ± 0.017

^a^ The value was given as the mean ± standard deviation (SD) based on the triplicate experiments.

## 3. Experimental Section

### 3.1. General Information

UV spectra were recorded on a V-530 spectrometer (JASCO, Tokyo, Japan). Measurements of electrospray ionization mass spectra were taken on an API-4000 instrument (AB Sciex, Framingham, MA, USA) and high-resolution fast atom bombardment-mass spectroscopy (HR-FAB-MS) was conducted on a JMS-700 MStation (JEOL, Tokyo, Japan) with a mixture of *m*-nitrobenzyl alcohol and dithiothreitol as the matrix. ^1^H and ^13^C-NMR spectra were recorded on an INOVA 600AS instrument (600 MHz for ^1^H and 151 MHz for ^13^C; Agilent, Santa Clara, CA, USA). Chemical shifts of the resonances in these spectra were adjusted using those of the solvent resonances [δ_H_ 2.04 and δ_C_ 29.8 for (CD_3_)_2_CO] and are given in δ (ppm) values. Analytical HPLC-DAD to monitor purification of the constituents was conducted on an ODS-A 302 (4.6 mm i.d. × 250 mm; YMC, Kyoto, Japan) column at 40 °C in an oven with 10 mM H_3_PO_4_/10 mM KH_2_PO_4_/MeCN (35:35:30, v/v/v, isocratic mode) as the eluent. A Hitachi L-2455 detector was used for monitoring UV absorption at 280 nm, and the flow rate was set at 1.0 mL/min. Preparative HPLC was performed on an ODS-A324 (10 mm i.d. × 300 mm: YMC) column at 40 °C in an oven with H_2_O/MeCN/MeCOOH (45:50:5, v/v/v) as the eluent. UV absorption at 280 nm was used for HPLC detection, and the flow rate was set at 2.0 mL/min. The procedure for the simultaneous HPLC analyses of the phenolic constituents in the EtOAc extract is described separately (see below). Silica gel (YMC), Toyopearl HW-40 (coarse grade; TOSOH, Tokyo, Japan), YMC-gel ODS-A (S, 75 μm; YMC), and MCI-gel CHP-20P (Mitsubishi Chemical, Tokyo, Japan) were used for column chromatography.

### 3.2. Plant Material

The crude drug used in this study was Tohoku licorice, which is the dried roots and stolons of *Glycyrrhiza uralensis* Fisch. ex DC, purchased from Tochimoto-tenkai-do (Osaka, Japan) (lot no. 002009037), and the GU-07112011(NEL) specimen was kept at the Medicinal Plant Garden, Okayama University Graduate School of Medicine, Dentistry and Pharmaceutical Sciences.

### 3.3. Extraction and Isolation

Licorice (1.0 kg) was pulverized and dipped in *n*-hexane (3 L × 2). Then, the defatted material was treated with EtOAc (3 L × 2) to give the extract (46.4 g). Part (40 g) of the EtOAc extract was subjected to column chromatography on ODS-gel (2.2 i.d. × 75 cm) with increasing concentrations of MeOH in H_2_O and then with increasing concentrations of CHCl_3_ in MeOH. The eluate with 50% CHCl_3_ in MeOH (3.6 g) was subjected to column chromatography on MCI-gel CHP-20P (2.2 i.d. × 45 cm) with increasing concentrations of MeOH in H_2_O. Fractions 94 (42 mg), 96 (40 mg), 161 (38 mg), 230 (31 mg), 231 (30 mg), 234 (28 mg), 327 (24 mg), and 337 (22 mg) were respectively purified by preparative HPLC on YMC-Pack A-324 (10 mm i.d. × 300 mm; 2.5 mL/min; H_2_O/MeCN/MeCOOH, 55:40:5, v/v/v; isocratic mode; monitored at 280 nm) to give the following phenolics: licoricidin (**2**, 3.1 mg), 7-*O*-methylluteone (**3**, 2 mg), and glyasperin J trimethyl ether (**4**, 1.5 mg) from fraction 94; 3'-(γ,γ-dimethylallyl)-kievitone (**5**, 4.0 mg), isoangustone A (**6**, 9.0 mg), glyasperin J (**7**, 2.3 mg), compound A (**8**, 3.2 mg), and licoriphenone (**9**, 1.9 mg) from fraction 96; demethylhomopterocarpan (**10**, 1.2 mg) from fraction 161; glycyrrhisofavone (**11**, 5.0 mg) from fraction 230; licopyranocoumarin (**12**, 4.9 mg), glyasperin C (**13**, 3.0 mg), and compound B (**14**, 2.0 mg) from fraction 231; glycyrrhiza-isofavone B (**15**, 1.6 mg) from fraction 234; glycybenzofuran (**16**, 1.8 mg) from fraction 327; and 8-(γ,γ-dimethylallyl)-wighteone (**17**, 1.5 mg) from fraction 337. The purity of each of the isolated compounds were >98%, as estimated by HPLC and ^1^H-NMR.

### 3.4. Spectral Data

Compound A (4'-*O*-Methylglycybenzofuran, **8**): This compound was obtained as a light brown powder; ^1^H- and ^13^C-NMR (see Table 1); HR-FAB-MS *m*/*z* 369.1702 ([M + H]^+^), (Calculated for C_22_H_24_O_5_, 369.1709).

Compound B (Neoglycybenzofuran, **14**): This compound was obtained as a light brown powder; ^1^H- and ^13^C-NMR (see Table 1); HR-FAB-MS *m*/*z* 355.1546 ([M + H]^+^), (Calculated for C_21_H_22_O_5_, 355.1549).

### 3.5. Methylation of Compounds A and B, and Glycybenzofuran

Trimethylsilyldiazomethane solution (1 mL) was added to a solution of **8** (1 mg) in EtOH (0.1 mL), and the mixture was kept for 3 h at room temperature. After evaporating the solvent, the remaining product was purified by TLC on silica gel (Merck, silica gel F254) (CHCl_3_‒MeOH, 15:1, v/v) to give a methyl derivative of compound A (**8a**). Detection was effected by UV absorption at 254 nm. ^1^H-NMR (600 MHz, acetone-*d*_6_): δ_H_ 1.99, 1.74, 1.65 (each 3H, s, –CH_3_ × 3), 3.27 (2H, d, *J* = 6.6 Hz, H-1"), 3.38, 3.82 (each 3H, s, -OCH_3_ × 2), 5.17 (1H, t, H-2"), 6.40 (1H, s, H-5'), 6.80 (1H, dd, *J* = 2.4, 8.4 H_Z_, H-5), 6.88 (1H, d, *J* = 2.4 H_Z_, H-7),7.35 (1H, d, *J* = 8.4 Hz, H-4). The identical compound was obtained by treating compound B (**14**) and glycybenzofuran (**16**) with trimethylsilyldiazomethane in analogous ways.

### 3.6. Antibacterial Assay

Estimations of the antibacterial effects of licorice phenolics on the VRE *E. faecium* FN-1 and *E. faecalis* NCTC 12201 used in this study were conducted using VRE kindly provided by Y. Ike, Gunma University. The bacterial cells were precultured in Mueller-Hinton broth at 37 °C under aerobic conditions. They were incubated in the presence of compounds with the concentrations obtained by serial two-fold dilution at 37 °C without shaking in the same broth for 24 h on microplates as shown in a previous paper [17], and their MICs were estimated as the lowest concentrations where the bacterial cells were not observed visually as reported previously [16,17], and were given based on triplicate experiments. DMSO was used for dissolving compounds hardly soluble in water, and the final concentrations were set at <1%, where DMSO has no effect. The positive control, linezolid, was dissolved in water.

### 3.7. Simultaneous HPLC Analysis of Phenolic Constituents in the EtOAc Extract of Licorice

Simultaneous analysis of licorice phenolics was carried out on an HPLC-DAD D-2000 HSM system, composed of an L-2130 pump (Hitachi, Tokyo, Japan) and an L-2455 DAD (Hitachi). The DAD was set for obtaining UV spectral data from 200 to 400 nm, and chromatograms at 280 nm were used for the quantitative analyses. The column used was an YMC-Pack pro C18 (6.0 mm i.d. × 150 mm) and was set in an oven at 40 °C. The mobile phase consisted of H_2_O/MeCN/MeCOOH (55:40:5, v/v/v), and the flow rate was set at 1.0 mL/min. Quantitation of **1**, **6**, **9**, **18**, **20**, **21**, **22**, and **25** was based on the HPLC profile monitored at 280 nm.

Licorice (10 g) was pulverized and extracted with EtOAc (100 mL × 3). Approximately 10 mg of the dried extract powder was dissolved in 10 mL of MeOH and filtered with a 0.45 µm PTFE membrane filter prior to injection (8 μL of the filtrate at 1 mg/mL) was applied to HPLC analysis. Stock solutions of eight licorice phenolics (**1**, **6**, **9**, **18**, **20**, **21**, **22** and **25**) were prepared at 0.1 mg/mL in MeOH, and diluted in series (from 0.1 to 0.001 mg/mL) to produce eight individual standard curves, for which the correlation coefficients were determined between 0.991 and 0.999 under the described HPLC conditions.

## 4. Conclusions

Our present investigation on the EtOAc extract of *G. uralensis* led to the purification of 16 compounds. Among the compounds obtained, two new compounds, **8** and **14**, had 2-aryl-3-methylbenzofuran structures, which rarely occur in Nature. The isolated phenolics were categorized into isoflavones (**3**, **6**, **11**, **15**, and **17**), isoflavans (**2** and **13**), isoflavanones (**4**, **5**, and **7**), a 3-arylcoumarin (**12**), a pterocarpan (**10**), 2-aryl-3-methylbenzofurans (**8**, **14**, and **16**), and a benzylphenylketone (**9**). As shown in our previous studies, licorice phenolics possess remarkable antibacterial effects against MRSA [16] and VRE [17]. The effects of the licorice phenolics isolated in the present study on VRE were examined. Based on their MIC values (Table 2), the antibacterial activities of the isoflavans and the isoflavones, bearing prenyl and phenolic hydroxyl groups, were promising. Our previous study [17] also indicated that compounds with prenyl moieties, such as gancaonin I (**1**), licoarylcoumarin (**19**), and glycycoumarin (**21**), showed noticeable anti-VRE effects. Taken together, we conclude that licorice phenolics, particularly those with prenyl moieties, could be used for the development of anti-VRE agents. The mechanisms of action of these phenolics as well as their potential synergistic effects remain to be clarified and the possibility of presence of potential synergistic effects between these identified licorice constituents are remained to be clarified. With regard to their promising antibiotic activities, the phenolic constituents from licorice could be used as lead compounds for developing new antibacterial agents.
